# Acupuncture of fascia points to relieve hand spasm after stroke: a study protocol for a multicenter randomized controlled trial

**DOI:** 10.1186/s13063-019-3999-7

**Published:** 2020-01-10

**Authors:** Zeng-Qiao Zhang, Kun-Peng Li, Jing He, Li-Ming Jiang, Wu Wang, Xiao-Shen Hu, Wei Feng

**Affiliations:** 1 Department of orthopedic rehabilitation, the Seventh People’s Hospital Affiliated to Shanghai University of Traditional Chinese Medicine, 358 Datong Road, Shanghai, 200137 China; 2 Department of Neurological Rehabilitation, the Second Rehabilitation Hospital of Shanghai, Shanghai, China; 3 School of Rehabilitation Science, Shanghai University of Traditional Chinese Medicine, Shanghai, China

**Keywords:** Fascia points, Acupuncture, Spasm, Stroke

## Abstract

**Background:**

The loss of functional ability of patients after stroke is mostly caused by dysfunction of the upper limbs, especially the hands. Hand functional exercise is the premise of alleviating hand dysfunction, and the relief of hand spasm is the basis of timely and effective hand functional exercise. Previous clinical observation have shown that fascial-point needling can effectively alleviate hand spasm immediately after stroke, but further evidence from large-sample studies is needed. The overall objective of this trial is to further evaluate the clinical efficacy of fascial-point acupuncture on hand spasm after stroke.

**Methods/design:**

This multicenter randomized controlled trial will compare the efficacy of fascial-point acupuncture versus sham acupuncture and routine rehabilitation therapy in stroke patients with hand spasm. Patients will be randomized to undergo either the fascial-point acupuncture, the sham acupuncture or the control (routine rehabilitation therapy). We will recruit 210 stroke inpatients who meet the trial criteria and observe the remission of hand spasm and improvement of limb function after 4 weeks of intervention. The first evaluation indices are the remission of hand spasm and the duration of spasm remission. The second evaluation indices are the hand function of the affected limbs and the activities of daily living. When the accumulative total number of cases included reaches 120, a mid-term analysis will be conducted to determine any evidence that experimental intervention does have an advantage.

**Discussion:**

Our aim is to evaluate the efficacy of fascial-point acupuncture in relieving hand spasm after stroke. The results should provide more evidence for the clinical application of this therapy in the future.

**Trial registration:**

Chinese Clinical Trial Registry (ChiCTR), ID: ChiCTR1900022379. Registered on 9 April 2019

## Background

With the advent of an aging society, the incidence of stroke is increasing year by year [1]. In recent years, due to the progress of science and technology and the continuous development of medicine, the survival rate and survival time of stroke patients have been greatly improved and prolonged, but the disability rate is still high [2]. The loss of activities of living of patients after stroke is mostly caused by upper-limb dysfunction, especially hand dysfunction [3]. Hand functional exercise is the premise of alleviating hand dysfunction, and the alleviation of hand spasm is the basis for timely and effective hand functional exercise. Hence, alleviating hand spasm after stroke has far-reaching significance in reducing disability rate and improving the daily activities of living of patients. At present, the main methods to relieve the increased muscle tension after stroke are drug intervention and non-drug therapy (such as physical factors and kinesiotherapy) [4, 5]. Sometimes, traditional Chinese medicine, acupuncture, massage, brace, orthosis and use of a rehabilitation robot are used as adjuvant therapy [6, 7]. Each therapy has its own advantages and disadvantages.

Drug intervention mainly includes using central antispasmodic drugs and peripheral-nerve local-blocking antispasmodic drugs. Current clinical applications have achieved some positive results. The use of central antispasmodic drugs is relatively simple and convenient, but long-term use will likely lead to obvious drug resistance and related adverse reactions, such as muscle weakness, nausea, mental depression, etc. In clinical application, drug replacement and dosage adjustment should be often considered. Botulinum toxin A is the representative of peripheral-nerve local-blockade antispasmodics. However, botulinum toxin A can only be used as a component of a multidisciplinary combination to alleviate the increase of limb-muscle tension after stroke. Other therapies are often needed in clinical application. In addition, in view of the technical difficulties and high cost of the clinical implementation of botulinum toxin A, it has not been widely carried out in the clinic to alleviate the increase of limb-muscle tension after stroke [8, 9]. Physical therapy is mainly divided into exercise therapy, manipulation therapy and physical factor therapy. Exercise therapy and manipulation therapy can inhibit and weaken spasticity-inducing factors in patients with post-stroke limb spasm, so that limb movement control and motor function can be significantly strengthened and improved. However, a longer intervening time should be guaranteed in the treatment of post-stroke limb spasticity, and the individual condition of the patients should be taken into account in clinical practice [10, 11]. In order to reduce the adverse effects caused by excessive exercise, we should adjust the range, intensity, frequency, and course of training. Physical factor therapy includes paraffin therapy, hydrotherapy, repetitive transcranial magnetic stimulation, biofeedback therapy, functional electrical stimulation and shock-wave therapy. Physical factor therapy has been widely used in the treatment of limb spasm after stroke, and has achieved certain effects, but its exact mechanism is still unclear, and there is a lack of evidence-based medical evidence for large-sample clinical research. In addition, the implementation of physical factor therapy has not yet been standardized or clinical guidelines set; the operation depends on personal preferences and experience, and the intensity of stimulation and dose in different clinical reports varies [12–16]. Orthosis and rehabilitation robots, with their good sustainability and rhythm, can assist in alleviating hand spasm after stroke and reduce the workload of therapists to some extent, but their sensitivity and regulation ability are poor, and they are expensive. They also require space and related technical personnel, which is not conducive with their clinical promotion [17, 18]. Although many positive results have been achieved in the study of traditional acupuncture and massage in the treatment of post-apoplectic limb spasm, it is often necessary to select multiple acupoints to achieve a curative effect. Also, the acupoints selected by each research group are different, which is not conducive with its clinical promotion [19]. Moreover, the criteria, principles and operating essentials of massage manipulation need to be unified and standardized, and its mechanism needs urgent clarification. All these circumstances encourage us to seek more simple, convenient, effective and inexpensive forms of rehabilitation therapy.

At present, the existence of myofascial trigger points has been widely accepted [20]. The myofascial trigger point is a common hand-spasm factor in stroke patients. In long-term rehabilitation clinical practice, the team found that physicians could touch a cord-shaped nodule as the most obvious sore point of the patient’s discomfort, i.e., the fascia point to be needled, by pressing between the first and second metacarpal bones on the dorsal of the patients with hand spasm after stroke from the far side to the proximal side with thumb pulp. Preliminary clinical observation of 16 patients with hand spasm after stroke treated by fascial-point acupuncture has been completed in our group. The results show that fascial-point acupuncture can effectively alleviate hand spasm immediately after stroke, but its cumulative effect, duration of spasm relief and long-term efficacy need further clinical research [21]. Therefore, we suggest that this multi-site, prospective clinical trial be carried out to further evaluate the clinical efficacy of fascial-point acupuncture in relieving hand spasm after stroke.

### Trial objectives

The objectives of this trial are as follows:

1. To verify the efficacy of fascial-point acupuncture in relieving hand spasm after stroke, and to improve the limb function and daily living ability of patients
2. To provide more evidence for the clinical application of this therapy in the future

## Methods/design

### Trial design

**Fig. 1:**
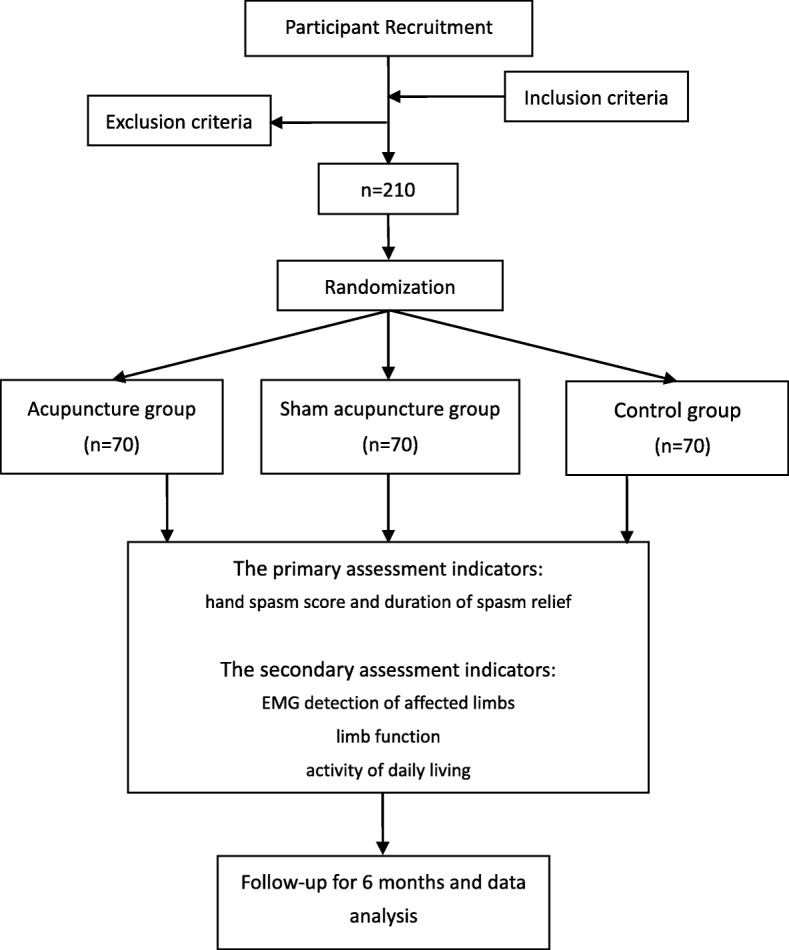
Flow chart of the study

**Fig. 2:**
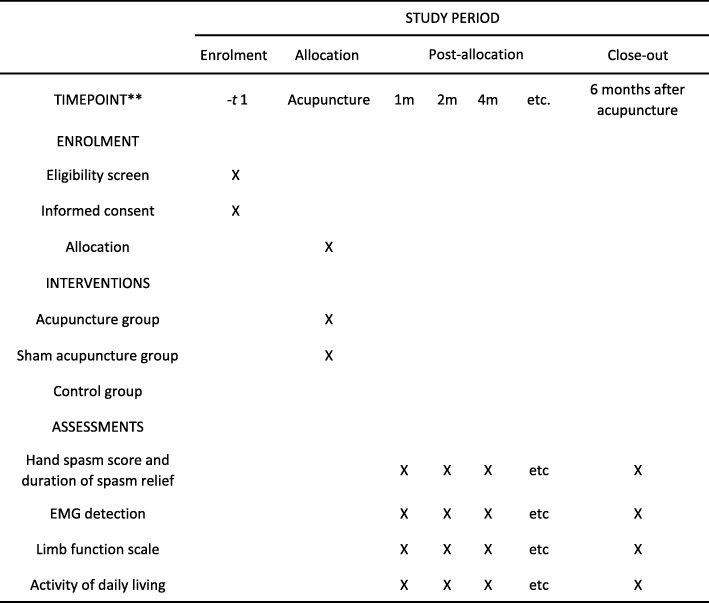
Example template of recommended content for the schedule, interventions, and assessments

This is a multicenter, prospective, randomized controlled trial supported by the Shanghai Science and Technology Commission. The trial will be carried out jointly by the Seventh People’s Hospital affiliated to Shanghai University of Traditional Chinese Medicine and two other hospitals in Shanghai. Patients meeting the pre-defined criteria will be randomly divided into three groups: an acupuncture group undergoing fascial-point acupuncture with routine rehabilitation treatment, a sham acupuncture group undergoing sham acupuncture near the fascial point with routine rehabilitation treatment, and a control group undergoing routine rehabilitation treatment. The patients will be followed up for 6 months to observe their hand spasm and limb function after treatment. The study flow chart is shown in Fig. 1. An example template for the content of admission plans, interventions and evaluation recommendations is shown in Fig. 2.

### Ethics

The Ethical Committee of the Seventh People’s Hospital affiliated to Shanghai University of Traditional Chinese Medicine approved the ethical approval of this study on 21 June, 2018 (reference number: 2018-IRBQYYS-012). The research scheme, patient information table and informed consent form were approved by the Ethics Committee. All participants will provide informed consent. The real names of the participants will not appear in the relevant reports of the trial to protect their privacy.

### Study setting

The research objects will be recruited from the Seventh People’s Hospital affiliated to Shanghai University of Traditional Chinese Medicine, Shanghai Second Rehabilitation Hospital and Shanghai Hudong Hospital. Interventions for all patients will be conducted in hospitals where participants are recruited. The Seventh People’s Hospital affiliated to Shanghai University of Traditional Chinese Medicine will be responsible for trial coordination and data management.

### Sample size

Our study will be designed as a randomized controlled trial, and the main outcome is whether hand spasm after stroke is relieved after treatment. Current experience shows that the previous effectiveness rate of conventional acupuncture treatment is about 50%, and the expected effectiveness rate is 85%. The significance test level is 0.05, and the test power is 0.9. Sample size is calculated by using:

$$ \mathit{\mathsf{N}}={\left(\mathit{\mathsf{U}\mathsf{\alpha }}+\mathit{\mathsf{U}\mathsf{\beta }}\right)}^{\mathsf{2}}\times \mathsf{2P}\left(\mathsf{1}-\mathit{\mathsf{P}}\right)/{\left(\mathit{\mathsf{P}\mathsf{1}}-\mathit{\mathsf{P}\mathsf{0}}\right)}^{\mathsf{2}}, $$

where, *N* is the required sample size for each treatment group, and the sample size of each group is equal. When *α* is 0.05 and *β* is 0.1, the normal distribution quantile table shows that:

$$ \mathit{\mathsf{U}\mathsf{\alpha }}\left(\mathsf{0.05}\right)=\mathsf{1.65},\mathsf{and} $$

$$ \mathit{\mathsf{U}\mathsf{\beta }}\left(\mathsf{0.1}\right)=\mathsf{1.28}. $$

*P0* and *P1* represent the original curative effect and the expected curative effect, 50% and 85%, respectively. By substituting the above parameters and values into the formulas, 63 cases will be needed for each group. Accounting for a 10% expulsion rate, the final estimated sample size will be about 70 cases per group (210 in total).

### Inclusion criteria

1. Cerebral hemorrhage or cerebral infarction confirmed by computed tomography (CT) or magnetic resonance imaging (MRI)
2. First onset, unilateral hemiplegia
3. The onset time is more than 2 weeks, and the vital signs are stable
4. Age 30–80 years
5. The clinical manifestations are spastic paralysis of the upper limbs, Brunnstrom stages II–IV of the upper limbs and hands with hemiplegia
6. The improved Ashworth score of the hand of the hemiplegic side is grade 1^+^ to 3
7. Stable condition, clear consciousness, no aphasia, no intellectual impairment, can understand the content of the scale and cooperate with the examination and treatment
8. No sedative or muscle relaxant has been taken in the last 2 weeks
9. Patients have signed an informed consent form

### Exclusion criteria

1. The patient’s condition in the critical or acute stage is unstable
2. Those with deafness, aphasia or severe cognitive impairment who find communicate difficult normally
3. Patients with psychiatric diseases, malignant tumors, severe bleeding tendency and infections of the treatment sites
4. Systolic blood pressure is more than 180 mmHg or diastolic blood pressure is more than 110 mmHg
5. Participating in other clinical trials or studies within 3 months and receiving other related treatments in the middle of the study may affect the judgment of the efficacy of this study
6. Dysfunction of muscle tone caused by other causes and previous motor dysfunction
7. Pregnant and lactating women
8. Fear of needling, fainting with needles, etc.

### Elimination criteria

1. Patients who have been mistakenly admitted or misdiagnosed
2. No intervention is given to the patients after admission

### Recruitment

Recruitment of patients began on 1 June 2019 and will be completed in June 2021, or after the required number is obtained, whichever is earlier. We will develop plans and methods to contact subjects, including the preparation of informed consent, obtaining necessary data, videos and pictures to help subjects understand the purpose and program of the trial. We will also explain to the subjects the advantages and disadvantages of the treatment and the relevant safety measures to be taken during the trial. In addition, we will post recruitment posters in and around the hospital, or use the Internet for recruitment. According to the inclusion/exclusion criteria, the research team will preliminarily judge the possibility of each subject’s inclusion, and conduct the relevant examination on them after they have signed the informed consent, and finally confirm whether the subjects actually meet the inclusion/exclusion criteria according to the examination results.

### Randomization

After signing the informed consent, participants will be randomly divided into three groups: an acupuncture group, a sham acupuncture group and a control group. Randomization will be accomplished by qualified researchers using randomization software to generate random-number sequences. In the process of randomization, allocation should be kept hidden. All patients will be randomly divided into groups in a ratio of 1:1:1. The strips revealing treatment allocation will be placed in sealed opaque envelopes with sequential numbers. After obtaining informed consent, the envelopes will be opened in turn. Patients and data analysts are not clear about the randomized grouping.

### Intervention

The interventions in the three groups are as follows:

1. Acupuncture group

In addition to routine rehabilitation treatment, fascial-point acupuncture will be given five times a week for 4 weeks, with 30 min each time.

**Fig. 3:**
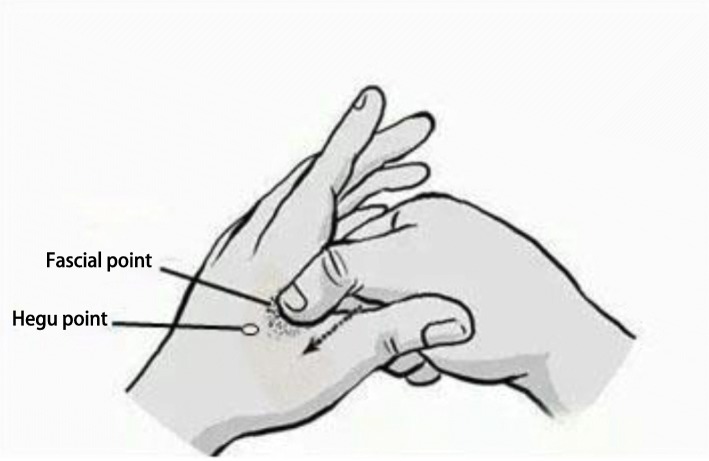
Location of fascial points

Location of fascial points: the patient will be in a sitting or supine position and the physician will be placed on the affected side. First, 75% alcohol cotton ball will be used to disinfect the patients´ dorsal between the first metacarpal bone and the second metacarpal bone and the thumb of the operator. Then, the operator will apply one-way pressure with thumb on the dorsal between the first metacarpal bone and the second metacarpal bone from the distal end to the closel end. During this process, the most obvious pain point or a cord nodule can be touched can be touched, which is the fascial point as shown in Fig. 3.

Acupuncture method: routine disinfection will be carried out on the hand of the operator and the relevant fascial point of the patient. According to individual differences of patients, different specifications and models of needles will be selected (25 mm–40 mm). Physicians quickly penetrate skin through the epidermis into the subcutaneous tissues about 1–2.5 cm with the needle tip vertical using a single-handed or two-handed needle-insertion method, and then through lifting, inserting and twisting, aim to enhance the needling sensation. The physician will stop manipulating the needle when there is a sense of needle ‘stagnation, described as being like “a fish swallowing a hook.” At the same time, pain and numbness will appear in the fascia area of the patient, and conduct to the fingers, resulting in tremor and convulsion of the hand.Pressing the needle hole with a dry, cotton-wool ball after needle removal is done to prevent bleeding. In the process of the needling operation, attention should be paid to coordinating the operation of both hands so as to achieve accuracy, rapidity of treatment, and painlessness or minimal pain.

2. Sham acupuncture group

In addition to routine rehabilitation treatment, treatment using false acupuncture beside the fascial points will be given five times a week for 4 weeks, with 30 min each time.

3. Control group

Routine rehabilitation treatment will be given five times a week for 4 weeks. Conventional rehabilitation treatment mainly includes:

- Good limb position: the affected upper limb maintains the position of abduction, external rotation, elbow extension, forearm supination, wrist and finger extension
- Bobath’s handshake exercises: the arm is raised over the head, and the mind is used to force the limbs on both sides 10 times a time for six times a day
- Exercise therapy: continuous stretching of spastic muscles and joint-loosening if necessary. The induced segregation movement and other manipulations are performed after relaxation of the spastic muscle, 45 min each time once a day

### Uniformity in acupuncture performance

To ensure the uniformity of fascial-point acupuncture, the coordinating center will designate qualified physicians to train physicians from other participating units and monitor the operation process to ensure qualified performance before the experiment.

### Outcome measures

Therapeutic evaluation will be carried out by the same team member who will be blinded to treatment allocation. The primary evaluation indicators in this study are the hand-spasm score and the duration of spasm relief. The modified Ashworth scale will be used to evaluate the spasm of the hand (mainly including the thumb and the other four fingers) before and after treatment. After the patients have been treated with acupuncture and spasticity relieved, the timing will be started to evaluate the duration of spasticity relieved (during this period, the patient should avoid mental tension, so as not to interfere with the test results).

The secondary evaluation indicators included using electromyography (EMG) to detect the activity of the affected limbs, limb function and activity of daily living evaluation. The modified Ashworth scale will be used to evaluate the degree of hand spasm on the affected side at baseline, 4 weeks after intervention and at 1, 2, 4 and 6 months of follow-up [22]. The modified Ashworth scale was divided into 0, 1, 1^+^, 2, 3 and 4 grades, and was quantified as 0, 1, 2, 3, and 4 and 5 points, respectively (Table 1). Surface EMG will be used to record the changes in surface EMG of the upper limbs on the affected side at baseline and 4 weeks after intervention [23]. The simplified Fugl-Meyer scale will be used to evaluate the upper-limb motor function on the affected side and the modified Barthel Index will be used to evaluate activities of daily living at baseline, 4 weeks after intervention and at 1, 2, 4 and 6 months of follow-up [24, 25]. All measurements will be recorded in the data center.

**Table 1:** Modified Ashworth scale

Grade	Assessment standard	Score
0	No increase in muscle tension	0
1	Muscle tension is increased slightly. When the affected part is passively flexed and stretched, it suddenly becomes stuck at the end of joint activity and then presents minimum resistance or release	1
1^+^	Muscle tension is increased slightly. Suddenly stuck in passive flexion and extension occurs within the last 50% of the range of ROM, and then shows minimal resistance	2
2	Muscle tension is increased significantly. Muscle tension of the affected limbs in passive motion increases significantly in most ROM ranges, but still easy to move	3
3	Muscle tension is increased severely. The affected limb’s passive movement has resistance over the whole ROM, so it is difficult to move	4
4	The affected part is rigid and inactive	5

*ROM* range of movement

### Harms

In our study, an adverse event will be defined as any untoward medical occurrence in a subject without regard to the possibility of a causal relationship. Adverse events will be collected after the subject has provided consent and enrolled in the study. If a subject experiences an adverse event after the informed consent document has been signed (entry) but the subject has not started to receive the study intervention, the event will be reported as not related to acupuncture. All adverse events occurring after entry into the study and until hospital discharge will be recorded. An adverse event that meets the criteria for a serious adverse event (SAE) between study enrollment and hospital discharge will be reported to the local Institutional Review Board.

### Data management

#### Data collection

All information should be truthfully, accurately and timely recorded in the case report form (CRF). Scale evaluators trained in rehabilitation will be responsible for assessing the simplified Fugl-Meyer scale, modified Ashworth scale and modified Barthel index, while other scales and case reports will be recorded by researchers. In the course of the experiment, special personnel will be requested to manage the relevant data, and the personal information of participants will be kept strictly confidential. All data will be identified using participant numbers, which do not directly display participant’s personal information. Data will not be shared without the explicit permission of the researchers. At the end of the trial, the research participants should submit the CRF in time and submit the test summary according to the requirements. The research center will appoint a supervisor to check the integrity and accuracy of the CRF.

#### Case report form

In the trial, the content recorded in the CRF should be consistent with the original material. The CRF must meet the following criteria:

1. Data must be entered with a black pen and signed
2. If the participant has received more than 2 weeks of intervention and evaluation, the data of the volunteer should still be recorded and counted
3. If an error occurs in the record and needs to be corrected, the recorder should draw a horizontal line under the original record, then sign the amendment and indicate the date of correction. Note that it must be ensured that the original record is identifiable after modification.

#### Database management and quality control

The team will take effective measures to control the quality. The data in the CRF will be entered into the database uniformly. Data-entry personnel carry out manual checks at the first time of data entry, and carry out systematic checks after all data entry has been completed. After final confirmation, the database is locked and saved. Any future changes to the database must be agreed in writing by the clinical research director, statistician and data administrator.

### Data analysis

#### Statistical analysis

Statistical analysis of research data will be performed by health statisticians and major researchers using SPSS or SAS. Pearson’s *χ*2 test or Fisher’s exact test will be used to analyze classified variables and continuous variables will be evaluated using Student’s *t* test or an appropriate non-parametric method. All statistical tests will be double-sided. The statistical significance level will be set at 5%. The measured data will be described by mean ± standard deviation. Before the analysis, the normality test and homogeneity test of variance will be carried out. If the normal distribution is satisfied, a *t* test will be used. The LSD or SNK method will be used for multiple comparisons, and the rank sum test will be used for non-normality or non-uniformity of variance.

## Discussion

The timely relief of spasticity in stroke patients is particularly important for the rehabilitation of limb function, so it is a key step to apply effective treatment measures to relieve spasticity as much as possible. A preliminary clinical observation of 16 cases of hand spasm after stroke treated by fascial-point acupuncture has been completed in our group. The results show that fascial-point acupuncture can effectively alleviate hand spasm immediately after stroke. However, its cumulative effect, duration of spasm relief and long-term efficacy need further clinical study. Hence, here we propose a multicenter, prospective and randomized clinical trial to further evaluate the clinical efficacy of fascial-point acupuncture in relieving hand spasm after stroke.

In order to eliminate the interference of acupuncture itself on the experimental results, a sham acupuncture group will be established in this study. However, bias exists in all clinical trials. In our trial, the blindness of the acupuncturists and patients in designing a randomized controlled trial involving acupuncture manipulation will be the most challenging aspect. Therefore, surface EMG measurements are also used as part of the outcome assessment in addition to the scales used in previous studies. In recent years, EMG has been widely used in spasticity assessment, especially after stroke [26]. The application of electrophysiological measurement can provide quantitative information of spasticity and reduce the interference of subjective factors in scale evaluation. In addition, the multicenter development of the study is also related to biases, mainly including differences in acupuncture techniques. We tried to eliminate these biases by examining the needling procedures of other participating hospitals and training acupuncturists in all participating centers. We firmly believe that the results of this study will help to lay a foundation for the alleviation of hand spasm after stroke and functional improvement.

## Trial status

The total registration period will last for 2 years and follow-up for 6 months. Recruitment of patients began on 1 June 2019, and trials are currently under way. Protocol version number and date: V1.0, 26 September 2019. Recruitment of patients is expected to be completed in October 2021.

## Supplementary information

**Additional file 1.** Standard Protocol Items: Recommendations for Interventional Trials (SPIRIT) 2013 Checklist [27]: recommended items to address in a clinical trial protocol and related documents.

## Data Availability

Data sharing is not applicable to this article as no datasets were generated or analyzed during the current study.
