# Supplementary material for: Acupuncture of fascia points to relieve hand spasm after stroke: a study protocol for a multicenter randomized controlled trial
Source: Trials. 2020 Jan 10;21:69. doi: 10.1186/s13063-019-3999-7 (PMC6954519; doi:10.1186/s13063-019-3999-7)
Supplement: Supplementary file 1 — Additional file 1. Standard Protocol Items: Recommendations for Interventional Trials (SPIRIT) 2013 Checklist [27]: recommended items to address in a clinical trial protocol and related documents. [file 13063_2019_3999_MOESM1_ESM.doc]

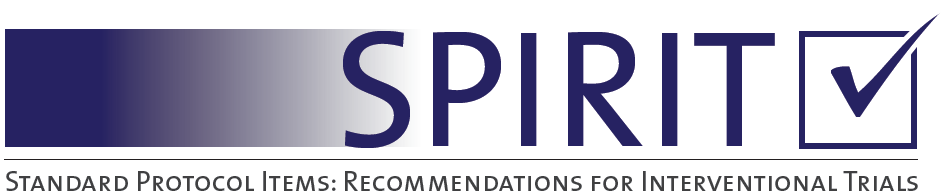


SPIRIT 2013 Checklist: Recommended items to address in a clinical trial protocol and related documents*

| Section/item | ItemNo | Description |
| --- | --- | --- |
| **Administrative information** | | |
| Title( Line 1) | 1 | A Multi-center, Evaluator-blinded, Randomized, 4-week Study to Evaluate the Efficacy of Fascial Point Acupuncture in Relieving Hand Spasm after Stroke. |
| Trial registration  ( Line 54) | 2a | Registration number：ChiCTR1900022379. |
|  | 2b | See the following record for details |
| Protocol version  ( Line 53) | 3 | The trial has been registered at the Chinese Clinical Trial Registry（ChiCTR）on April 9, 2019. No modifications have been made. |
| Funding  ( Line 363) | 4 | This study was supported by Traditional Chinese Medicine Guidance Project of Shanghai Science and Technology Commission [grant number 18401900300]. |
| Roles and responsibilities  ( Line 358) | 5a | Zeng-Qiao Zhang, Kun-Peng Li, Jing He, Li-Ming Jiang, Wu Wang, Xiao-Shen Hu and Wei Feng. FW conceives and designs this research, and is the person in charge of this research. ZZQ and LKP were the main implementers of the study and drafted manuscripts. HJ, JLM, WW and HXS participated in the design of the study and assisted in drafting the manuscript. All authors know and agree with the final manuscript. |
|  | 5b | Trial Sponsor: Shanghai Science and Technology Commission  Address: 200 Renmin Avenue, Shanghai  Telephone: 23111111  Email: jiandu@stcsm.gov.cn |
|  | 5c | This funding source had no role in the design of this study and will not have any role during its execution, analyses, interpretation of the data, or decision to submit results |
|  | 5d | Principal Investigator and Research Physician  Design and conduct of RITUXVAS  Preparation of protocol and revisions  Preparation of investigators brochure (IB) and CRFs [Case Report Forms]  Organising steering committee meetings  Managing CTO [Clinical Trials Office]  Publication of study reports  Members of TMC [Trial Management Committee]  Steering committee (SC)  (see title page for members)  Agreement of final protocol  All lead investigators will be steering committee members. One lead investigator per country will be nominated as national, coordinator.  Recruitment of patients and liasing with principle [sic] investigator  Reviewing progress of study and if necessary agreeing changes to the protocol and/or investigators brochure to facilitate the smooth running of the study.  Trial Management Committee (TMC)  (Principle [sic] investigator, Research Physician, Administrator)  Study planning  Organisation of steering committee meetings  Provide annual risk report MHRA [Medicines and Healthcare products Regulatory Agency] and ethics committee  SUSAR [Serious unexpected suspected adverse events] reporting to MHRA and Roche  Responsible for trial master file  Budget administration and contractual issues with individual centres  Advice for lead investigators  Audit of 6 monthly feedback forms and decide when site visit to occur.  Assistance with international review, board/independent ethics committee applications  Data verification  Randomisation  Organisation of central serum sample collection  Data Manager  Maintenance of trial IT system and data entry  Data verification  Lead Investigators  In each participating centre a lead investigator will be identified, to be responsible for identification, recruitment, data collection and completion of CRFs, along with follow up of study patients and adherence to study protocol and investigators brochure . . . Lead investigators will be steering committee members, with one investigator per country being nominated as national coordinator.” |
| Introduction |  |  |
| Background and rationale  ( Line 64) | 6a | The loss of life ability of patients after stroke is mostly caused by upper limb dysfunction, especially hand dysfunction. Hand functional exercise is the premise to alleviate hand dysfunction, and the alleviation of hand spasm is the basis for timely and effective hand functional exercise. Therefore, alleviating hand spasm after stroke has far-reaching significance in reducing disability rate and improving daily living ability of patients. At present, the main methods to relieve the increased muscle tension after stroke are drug intervention and non-drug therapy (such as physical factors and kinesiotherapy). Sometimes, traditional Chinese medicine, acupuncture, massage, brace, orthosis and rehabilitation robot are used as adjuvant therapy. Each therapy has its own advantages and disadvantages.  Drug intervention mainly includes central antispasmodic drugs and peripheral nerve local blocking antispasmodic drugs. Current clinical applications have also achieved certain results. In clinical application, drug replacement and dosage adjustment should be often considered. Physical therapy is mainly divided into exercise therapy, manipulation therapy and physical factor therapy. The operation depends on personal preferences and experience, the intensity of stimulation and dose of clinical reports are different. Orthosis and rehabilitation robots, with their good sustainability and rhythm, can assist in alleviating hand spasm after stroke and reduce the workload of therapists to some extent, but their sensitivity and regulation ability are poor, and their price is expensive. They also require space and related technical personnel, which is not conducive to clinical promotion. Although many positive results have been reported in the study of traditional acupuncture and massage therapy to reduce the muscle tension of spastic limbs after stroke, the selection of acupuncture points is too extensive, lacking of internal links and inconsistent syndromes, which is not conducive to summary and clinical promotion. All these circumstances encourage us to seek more simple, convenient, effective and inexpensive rehabilitation therapy. |
|  | 6b | Myofascial trigger point is a common hand spasm factor in stroke patients. In the long-term rehabilitation clinical practice, the team found that doctors could touch a cord-shaped nodule or the most obvious soreness point of the patient's sensation, i.e. the fascia point to be needled, by pressing between the first and second metacarpal bones on the dorsal palm of the patients with hand spasm after stroke from the far side to the proximal side with thumb pulp. Preliminary clinical observation of 16 patients with hand spasm after stroke treated by fascial point acupuncture has been completed in our group. The results show that fascial point acupuncture can effectively alleviate hand spasm immediately after stroke, but its cumulative effect, duration of spasm relief and long-term efficacy need further clinical research. Therefore, we suggest that this multi-site, prospective clinical trial be carried out to further evaluate the clinical efficacy of fascial point acupuncture in relieving hand spasm after stroke. |
| Objectives  ( Line 127) | 7 | The objectives of this trial are as follows:  1. To verify the efficacy of fascial point acupuncture in relieving hand spasm after stroke, and to improve the limb function and daily living ability of patients.  2. To provide more evidences for the clinical application of this therapy in the future. |
| Trial design  ( Line 133) | 8 | This is a multi-center, prospective randomized controlled trial supported by Shanghai Science and Technology Commission. All patients will be randomly divided into groups according to the ratio of 1:1:1. The strips revealing treatment allocation are placed in sealed opaque envelopes with sequential numbers. After obtaining informed consent, the envelopes will be opened in turn. Patients and data analysts are not clear about the randomized grouping. |
| Methods: Participants, interventions, and outcomes | | |
| Study setting  ( Line 154) | 9 | The trial will be carried out jointly by the Seventh People's Hospital affiliated to Shanghai University of Traditional Chinese Medicine and two other hospitals in Shanghai. |
| Eligibility criteria  ( Line 172) | 10 | Inclusion criteria:  (1) Cerebral hemorrhage or cerebral infarction confirmed by CT or MRI;  (2) First onset, unilateral hemiplegia;  (3) The onset time is more than 2 weeks, and the vital signs are stable;  (4) Age 30-80 years old;  (5) The clinical manifestations are spastic paralysis of upper limbs, Brunnstrom stage II-IV of upper limbs and hands with hemiplegia;  (6) The improved Ashworth score of hemiplegic side hand is 1+ - 3 grade;  (7) Stable condition, clear consciousness, no aphasia, no intellectual impairment, can understand the content of the scale and cooperate with the examination and treatment;  (8) No sedative or muscle relaxant is taken in 2 weeks;  (9) Patients have signed informed consent forms.  Exclusion criteria:  (1) The condition in critical or acute stage is not stable;  (2)Those with deafness, aphasia or severe cognitive impairment who are difficult to communicate normally;  (3) Patients with psychiatric diseases, malignant tumors, severe bleeding tendency and infections of treatment sites;  (4) Systolic blood pressure is more than 180 mmHg or diastolic blood pressure is more than 110 mmHg;  (5) Participating in other clinical trials or studies within 3 months and receiving other related treatments in the middle of the study may affect the judgement of the efficacy of this study;  (6) Dysfunction of muscle tone caused by other causes and previous motor dysfunction;  (7) Pregnant and lactating women;  (8) Fear of needling, fainting needles, etc. |
| Interventions  ( Line 214) | 11a | The interventions in the three groups are as follows:  Acupuncture group  On the basis of routine rehabilitation treatment, fascial point acupuncture will be given 5 times a week for 4 weeks, with 30 minutes each time.  Location of fascial points: the patient is in a sitting or supine position and the doctor is placed on the affected side. Firstly, 75% alcohol cotton ball is used to routinely disinfect the area of the the first web and the finger of the operator. Then, one-way pressure is applied between the first and second metacarpal bones on the dorsal palm of the patient from the far side to the proximal side with the thumb pulp. At this time, a cord-shaped nodule can be touched. Or the patient feels the most obvious soreness point, which is the fascial point. As shown in Figure 3.  Acupuncture method: routine disinfection is carried out on the hand of the operator and the fascial spot area of the patient. According to individual differences of patients, different specifications and models of needles are selected. Doctors quickly penetrate the needle tip vertically through the epidermis into the subcutaneous about 0.5-1.0 inches by using single-handed or two-handed needle insertion method, and then through lifting, inserting and twisting to enhance the sense of needle. When the doctor feels a slight sense of needle stagnation, such as fish swallowing hook. At the same time, the patient's fascial point area will be sore, numb and painful, accompanied by finger conduction pain and tremor and convulsion. Press the needle hole with dry cotton ball after needle discharge to prevent bleeding. In the process of needling operation, attention should be paid to the coordinated operation of both hands so as to achieve accurate, rapid, painless or less pain.  Sham acupuncture group  On the basis of routine rehabilitation treatment, treatment of false acupuncture beside Fascial Points will be given 5 times a week for 4 weeks, with 30 minutes each time.  Control group  Routine rehabilitation treatment will be given 5 times a week for 4 weeks. Conventional rehabilitation treatment mainly includes:  (1) Good limb position: the affected upper limb maintains the position of abduction, external rotation, elbow extension, forearm supination, wrist and finger extension;  (2) Bobath's handshake exercises: the arm is raised over the head, and the mind is used to force the limbs on both sides 10 times a time for 6 times a day;  (3) Exercise therapy: Continuous pulling of spastic muscle and joint loosening if necessary. The induced segregation movement and other manipulations are performed after the relaxation of the spastic muscle, 45 minutes each time for once a day. |
|  | 11b | Elimination criteria  (1) Patients who have been mistakenly admitted or misdiagnosed;  (2) No intervention is given to the patients after admission. |
|  | 11c | Before fascial point acupuncture treatment, patients and their families should be informed of the treatment purpose, treatment risk, precautions after treatment, possible complications and preventive measures. |
|  | 11d | Common adverse events of acupuncture and treatment methods should be well known before treatment. |
| Outcomes  ( Line 254) | 12 | The primary evaluation indicators in this study are hand spasm score and duration of spasm relief. The secondary evaluation indicators included EMG detection of affected limbs, limb function and activity of daily living evaluation. |
| Participant timeline  ( Line 254) | 13 | Treatment will be given 5 times a week for 4 weeks, with 30 minutes each time. Outcomes will be measured at baseline, four weeks after intervention and at 1, 2, 4 and 6 months of follow-up. |
| Sample size  ( Line 160) | 14 | Our study will be designed as a randomized controlled trial, and the main outcome is whether hand spasm after stroke is relieved after treatment. Current experience shows that the previou effective rate of conventional acupuncture treatment is about 50%, and the expected effective rate is 85%. The significance test level was 0.05, and the test power was 0.9. Sample size was calculated by using N =（Uα＋Uβ）²*2P（1－P）/（P1－P0）². Among them, N was the required sample size for each treatment group, and the sample size of each group was equal. When α was 0.05 and β was 0.1, the normal distribution quantile table shows that: Uα(0.05) = 1.65, Uβ(0.1) = 1.28; P0 and P1 represent the original curative effect and the expected curative effect, 50% and 85% respectively. By substituting the above parameters and values into the formulas, 63 cases were needed for each group.Accounting for a 10% expulsion rate, the final estimated sample size was about 70 cases per group (210 in total). |
| Recruitment  ( Line 200) | 15 | Recruitment of patients began on 1 June 2019 and will be completed in June 2021, or after the required number is obtained, whichever is earlier. Patients who meet the criteria will be invited to participate in the trial, and researchers will explain to them the relevant issues in the course of the trial. |
| **Methods: Assignment of interventions (for controlled trials)** | | |
| Allocation: |  |  |
| Sequence generation  ( Line205) | 16a | Random numbers were generated by computer .All patients will be randomly divided into groups according to the ratio of 1:1:1. |
| Allocation concealment mechanism  ( Line 205) | 16b | The strips revealing treatment allocation are placed in sealed opaque envelopes with sequential numbers. After obtaining informed consent, the envelopes will be opened in turn. Patients and data analysts are not clear about the randomized grouping. |
| Implementation  ( Line 205) | 16c | All patients who give consent for participation and who fulfil the inclusion criteria will be randomized. Randomisation will be requested by the staff member responsible for recruitment and clinical interviews from CenTrial [Coordination Centre of Clinical Trials].  In return, CenTrial will send an answer form to the study therapist who is not involved in assessing outcome of the study. This form will include a randomisation number. In every centre closed envelopes with printed randomisation numbers on it are available. For every randomisation number the corresponding code for the therapy group of the randomisation list will be found inside the envelopes. The therapist will open the envelope and will find the treatment condition to be conducted in this patient. The therapist then gives the information about treatment allocation to the patient. Staff responsible for recruitment and symptom ratings is not allowed to receive information about the group allocation.  The allocation sequence will be generated by the Institute for Medical Biometry (IMB) applying a permuted block design with random blocks stratified by study centre and medication compliance (favourable vs. unfavourable) . . . The block size will be concealed until the primary endpoint will be analysed. Throughout the study, the randomisation will be conducted by CenTrial in order to keep the data management and the statistician blind against the study condition as long as the data bank is open. The randomisation list remains with CenTrial for the whole duration of the study. Thus, randomisation will be conducted without any influence of the principal investigators, raters or therapists.” |
| Blinding (masking)  ( Line 255) | 17a | Assessments regarding clinical recovery will be conducted by an assessor blind to treatment allocation. The assessor will go through a profound assessment training program . Due to the nature of the intervention neither participants nor staff can be blinded to allocation, but are strongly inculcated not to disclose the allocation status of the participant at the follow up assessments. An employee outside the research team will feed data into the computer in separate datasheets so that the researchers can analyse data without having access to information about the allocation |
|  | 17b | To maintain the overall quality and legitimacy of the clinical trial, code breaks should occur only in exceptional circumstances when knowledge of the actual treatment is absolutely essential for further management of the patient. Investigators are encouraged to discuss with the Medical Advisor or PHRI [Population Health Research Institute] physician if he/she believes that unblinding is necessary.  If unblinding is deemed to be necessary, the investigator should use the system for emergency unblinding through the PHRI toll-free help line as the main system or through the local emergency number as the back-up system.  The Investigator is encouraged to maintain the blind as far as possible. The actual allocation must NOT be disclosed to the patient and/or other study personnel including other site personnel, monitors, corporate sponsors or project office staff; nor should there be any written or verbal disclosure of the code in any of the corresponding patient documents.  The Investigator must report all code breaks (with reason) as they occur on the corresponding CRF [case report form] page. |
| **Methods: Data collection, management, and analysis** | | |
| Data collection methods  ( Line 278) | 18a | Therapeutic evaluation will be carried out by the same team member without knowing the patients'grouping and observation time point. The primary evaluation indicators in this study are hand spasm score and duration of spasm relief. The secondary evaluation indicators included EMG detection of affected limbs, limb function and activity of daily living evaluation. The modified Ashworth scale will be used to evaluate the degree of hand spasm on the affected side at baseline, four weeks after intervention and at 1, 2, 4 and 6 months of follow-up. The modified Ashworth scale was divided into 0, 1, 1 ^+^, 2, 3 and 4 grades, and was quantified as 0, 1, 2, 3, 4 and 5 points respectively. Surface electromyography will be used to record the changes of surface electromyography of upper limbs on the affected side at baseline and four weeks after intervention. Simplified Fugl-Meyer scale will be used to evaluate the upper limb motor function on the affected side and modified barthel index will be used to evaluate activities of daily living at baseline, four weeks after intervention and at 1, 2, 4 and 6 months of follow-up. All measurements will be recorded in the data center. |
|  | 18b | All information should be truthfully, accurately and timely recorded in the case report form (CRF). Scale evaluators trained in rehabilitation will be responsible for assessing the simplified Fugl-Meyer scale, Modified Ashworth scale and Modified Barthel index, while other scales and case reports will be recorded by researchers. In the course of the experiment, special personnel will be arranged to manage the relevant data, and the personal information of participants will be kept strictly confidential. All data will be named using participant numbers, which do not directly display participant's personal information. Data will not be shared without the explicit permission of researchers. At the end of the trial, the research participants should submit the case record form in time and submit the test summary according to the requirements. The research center will appoint a supervisor to check the integrity and accuracy of CRF. Statisticians are responsible for the analysis and summary of all the data submitted, and we finally come to a conclusion. |
| Data management  ( Line 299) | 19 | The team will take effective measures to control the quality. Data in CRF will be entered into the database uniformly. Data entry personnel carry out manual checks at the first time of data entry, and carry out systematic checks after all data entry is completed. After final confirmation, the database is locked and saved. Any future changes to the database must be agreed in writing by the clinical research director, statistician and data administrator. |
| Statistical methods  ( Line 318) | 20a | Statistical analysis of research data is performed by health statisticians and major researchers using SPSS or SAS. Pearson’s χ2 test or Fisher’s exact test will be used to analyze classified variables and continuous variables will be evaluated by Student’s t-test or an appropriate non-parametric method. All statistical tests will be double-sided. Statistical significance level will be set at 5%. The measured data will be described by mean±standard deviation. Before the analysis, the normality test and homogeneity test of variance are carried out. If the normal distribution is satisfied, t test is used. LSD or SNK method is used for multiple comparisons, and rank sum test is used for non-normality or non-uniformity of variance. |
|  | 20b | No additional analysis |
|  | 20c | Nevertheless, we propose to test non-inferiority using two analysis sets; the intention-to-treat set, considering all patients as randomized regardless of whether they received the randomized treatment, and the “per protocol” analysis set. Criteria for determining the “per protocol” group assignment would be established by the Steering Committee and approved by the PSMB [Performance and Safety Monitoring Board] before the trial begins. Given our expectation that very few patients will crossover or be lost to follow-up, these analyses should agree very closely. analysis sets.  We will report reasons for withdrawal for each randomization group and compare the reasons qualitatively. The effect that any missing data might have on results will be assessed via sensitivity analysis of augmented data sets. Dropouts (essentially, participants who withdraw consent for continued follow-up) will be included in the analysis by modern imputation methods for missing data.  The main feature of the approach is the creation of a set of clinically reasonable imputations for the respective outcome for each dropout. This will be accomplished using a set of repeated imputations created by predictive models based on the majority of participants with complete data. The imputation models will reflect uncertainty in the modeling process and inherent variability in patient outcomes, as reflected in the complete data. |
| **Methods: Monitoring** | | |
| Data monitoring  ( Line 306) | 21a | A Data Monitoring Committee (DMC) has been established. The DMC is independent of the study organisers. During the period of recruitment to the study, interim analyses will be supplied, in strict confidence, to the DMC, together with any other analyses that the committee may request. This may include analyses of data from other comparable trials. |
|  | 21b | An interim-analysis is performed on the primary endpoint when 50% of patients have been randomised and have completed the 6 months follow-up. The interim-analysis is performed by an independent statistician, blinded for the treatment allocation. The statistician will report to the independent DSMC [data and safety monitoring committee]. The DSMC will have unblinded access to all data and will discuss the results of the interim-analysis with the steering committee in a joint meeting. The steering committee decides on the continuation of the trial and will report to the central ethics committee. The Peto approach is used: the trial will be ended using symmetric stopping boundaries at P < 0.001 [Reference X]. The trial will not be stopped in case of futility, unless the DSMC during the course of safety monitoring advices [sic] otherwise. In this case DSMC will discuss potential stopping for futility with the trial steering committee. |
| Harms  ( Line 268) | 22 | In our study an adverse event will be defined as any untoward medical occurrence in a subject without regard to the possibility of a causal relationship. Adverse events will be collected after the subject has provided consent and enrolled in the study. If a subject experiences an adverse event after the informed consent document is signed (entry) but the subject has not started to receive study intervention, the event will be reported as not related to acupuncture. All adverse events occurring after entry into the study and until hospital discharge will be recorded. An adverse event that meets the criteria for a serious adverse event (SAE) between study enrollment and hospital discharge will be reported to the local IRB [Institutional Review Board] as an SAE. |
| Auditing  ( Line 299) | 23 | Through the combination of our web-based, instantaneous electronic validation, the DCC’s [Data Coordinating Center] daily visual cross-validation of the data for complex errors, and regular on-site monitoring, the quality and completeness of the data will be reflective of the state of the art in clinical trials. |
| Ethics and dissemination | | |
| Research ethics approval  ( Line 290) | 24 | Subsequent to initial review and approval, the responsible local Institutional Review Boards/Ethical Committees (IRBs/ECs) will review the protocol at least annually. The Investigator will make safety and progress reports to the IRBs/ECs at least annually and within three months of study termination or completion at his/her site. These reports will include the total number of participants enrolled and summaries of each DSMB [Data Safety and Monitoring Board] review of safety and/or efficacy. |
| Protocol amendments  ( Line 369) | 25 | Any modifications to the protocol which may impact on the conduct of the study, potential benefit of the patient or may affect patient safety, including changes of study objectives, study design, patient population, sample sizes, study procedures, or significant administrative aspects will require a formal amendment to the protocol. |
| Consent or assent  ( Line 369) | 26a | Trained Research Nurses will introduce the trial to patients who will be shown a video regarding the main aspects of the trial. Patients will also receive information sheets. Research Nurses will discuss the trial with patients in light of the information provided in the video and information sheets. Patients will then be able to have an informed discussion with the participating consultant. Research Nurses will obtain written consent from patients willing to participate in the trial. |
|  | 26b | No |
| Confidentiality  ( Line 299) | 27 | All study-related information will be stored securely at the study site. All participant information will be stored in locked file cabinets in areas with limited access. All laboratory specimens, reports, data collection, process, and administrative forms will be identified by a coded ID [identification] number only to maintain participant confidentiality. All records that contain names or other personal identifiers, such as locator forms and informed consent forms, will be stored separately from study records identified by code number. All local databases will be secured with password-protected access systems. Forms, lists, logbooks, appointment books, and any other listings that link participant ID numbers to other identifying information will be stored in a separate, locked file in an area with limited access.Participants’ study information will not be released outside of the study without the written permission of the participant. |
| Declaration of interests  ( Line 376) | 28 | There were no financial and other competing interests. |
| Access to data  ( Line 366) | 29 | The Data Management Coordinating Center will oversee the intra-study data sharing process, with input from the Data Management Subcommittee.All Principal Investigators will be given access to the data sets. |
| Ancillary and post-trial care  ( Line 268) | 30 | Patients that are enrolled into the study are covered by indemnity for negligent harm through the standard NHS [National Health Service] Indemnity arrangements. |
| Dissemination policy  ( Line 290) | 31a | The Publications subcommittee will review all publications following the guidelines given below and report its recommendations to the Steering Committee |
|  | 31b | Topics suggested for presentation or publication will be circulated to the PIs [Principal investigators] of the CCCs [Core Coordinating Centers], the DCC [Data Coordinating Center], Core Lab and the NIH [National Institutes of Health]. These groups are requested to suggest and justify names for authors to be reviewed by the PC [Publications Committee]. |
|  | 31c | Data sharing statement No later than 3 years after the collection of the 1-year postrandomisation interviews, we will deliver a completely deidentified data set to an appropriate data archive for sharing purposes. |
| Appendices |  |  |
| Informed consent materials | 32 | Model consent form and other related documentation given to participants and authorised surrogates |
| Biological specimens | 33 | No |

*It is strongly recommended that this checklist be read in conjunction with the SPIRIT 2013 Explanation & Elaboration for important clarification on the items. Amendments to the protocol should be tracked and dated. The SPIRIT checklist is copyrighted by the SPIRIT Group under the Creative Commons “[Attribution-NonCommercial-NoDerivs 3.0 Unported](http://www.creativecommons.org/licenses/by-nc-nd/3.0/)” license.
